# A concise review of dental sealants in caries management

**DOI:** 10.3389/froh.2023.1180405

**Published:** 2023-04-17

**Authors:** Toby Cheuk-Hang Ng, Chun-Hung Chu, Ollie Yiru Yu

**Affiliations:** Faculty of Dentistry, The University of Hong Kong, Hong Kong SAR, China

**Keywords:** dental sealant, dental caries, caries management, cariology, preventive dentistry, oral health

## Abstract

This concise review provides an update of the use of dental sealants. Dental sealants protect a tooth from caries development by providing a physical barrier to microorganism colonisation and creating a favorable environment for patients to clean. Some sealants release fluoride ions to promote remineralization. Dental sealants can be applied on pits and fissures of primary and permanent teeth to prevent and arrest early enamel caries. They are effective in caries prevention. The preventive fraction of resin sealant is up to 61% after 5 years. Dental sealants can be classified as resin, glass ionomer and hybrid (compomer or giomer) according to the material. Recent studies from 2012 to 2022 showed resin sealant has a high retention rate of up to 80% after 2 years, whereas glass ionomer sealants is 44%. Chemical etching with 37% phosphoric acid is the standard of care, whereas laser or air-abrasion cannot does not increase the sealant retention rate. Moist control is critical and studies found the use of rubber dam and cotton rolls had similar success for sealant retention. The longevity of dental sealant is also associated with clinical operative factors, including approaches of moisture control, enamel pretreatment, selection of dental adhesive, and time of acid etching.

## 1. Introduction

For Dental caries, commonly known as tooth decay, is a highly prevalent oral disease around the world. More than 2.4 billion people are suffering from permanent teeth caries while 520 million children are affected by primary teeth caries (1). Untreated dental caries gradually extends and irritate the dental pulp, leading to the destruction of the tooth structure, excruciating pain, and eventually tooth extraction (2). Dental caries degrades the quality of life of the patients (3). It can also lead to lower self-esteem (4) and depression (5). Pits and fissure areas in the tooth surface are highly susceptible to dental caries. Dental caries was the demineralization of dental hard tissue caused by acid produced by the fermentation of carbohydrates by cariogenic bacteria in the dental plaque biofilm (6). Because of the anatomical structure, the dental plaque biofilm in the fissure is difficult, if not impossible, to remove through daily oral hygiene practice. The caries risk is higher in teeth with deep fissures (7, 8). An observational study found caries were located in the occlusal fissure areas in 79% of the decayed molars (9).

Because of the susceptibility of pit and fissure areas to dental caries, oral care providers have been trying to eliminate fissures to control caries. The initial approach to eliminate fissures was invasive. Hyatt suggested prophylactic odontotomy in 1923, which was an invasive dental procedure to remove deep fissures with a dental bur and place a filling to prevent caries (10). Three decades later in 1955, Buonocore proposed a fissure sealing procedure with the 85% phosphoric acid etching of enamel followed by a resin material coverage on the fissure area (11). This technique was a foundation for the further development of fissure sealing using different dental materials. Cueto suggested using methyl cyanoacrylate to seal pits and fissures in 1965 (12). Later, Buonocore successfully applied light-cure dental sealant incorporated with Bisphenol A-glycidyl methacrylate (Bis-GMA) in 1970 (13). In the following year, the Council on Dental Materials Devices and Council on Dental Therapeutics of the American Dental Association recognized the use of dental sealant (14). The first glass ionomer sealant appeared in the mid-1970s (15).

As a common preventive strategy for dental caries, the understanding and the application of dental sealant has been shifting in accordance with the contemporary paradigm of caries management, which placed prevention as a priority (16). The clinical application of dental sealant has been broadening when compared to the past. Dental sealant was majorly used in the pits and fissure area of the occlusal surface in the posterior area in the past. It is now a common preventive measure to manage dental caries in the occlusal or approximal surfaces of the anterior or posterior teeth in primary or permanent dentition. It can also be used on exposed root surfaces to prevent root caries. In addition, with the development of dental material sciences, more materials are available as dental sealants. Therefore, the objective of this review is to overview and update the knowledge of dental sealants in the aspect of their classification, the mechanism in caries prevention, clinical indications, effectiveness in caries control, clinical longevity and factors affecting the clinical outcomes in the 2012–2022.

## 2. Literature search

Keywords ((fissure sealant) OR (dental sealant)) AND (dental caries) are used to search for articles in the PubMed and Scopus databases. Articles published from 2012 to 2022 were selected. After duplicate removal, 886 articles were identified. The titles and abstracts of the identified article were screened. Finally, we included 175 articles on dental sealants and caries for this review. Among the included articles, 27 articles were randomized clinical trials comparing caries prevention and/or retention rate of dental sealant.

## 3. Classification of dental sealant by material

Dental sealants can be broadly classified into three categories based on the components—resin sealant, glass ionomer sealant and hybrid sealant.

### 3.1. Resin sealant

Resin sealant contains an organic resin-based oligomer matrix (17). Bis-GMA is the most common monomer for the matrix of resin sealant. Other resin monomers such as Urethane-dimethacrylate (UDMA) and Triethylene glycol dimethacrylate (TEGDMA) are in alternative use (18). Resin sealant may also contain filler, fluoride particles or photoinitiations.

#### 3.1.1. Auto-cure resin sealant or light-cure resin sealant

Based on the ways of polymerization, resin sealant could be further classified as self-cure or light-cured resin sealant. Resin sealant sets after undergoing polymerisation of monomers. The polymerisation occurs in two ways, auto-polymerisation or polymerisation under an external light source. The setting of self-cured resin sealant is an auto-polymerisation reaction initiated by chemical reactions. The chemical reactions of auto-polymerisation process are initiated by the free radicals produced by chemical reactions of tertiary amine and benzoyl peroxide (19). No external light source is required. Light-cured resin sealant requires an external light source to imitate the polymerisation. Photo-initiators in the resin sealant absorb light radiation, dissociating into free radicals, and start the polymerisation process (20). The external light source could be ultraviolet or visible light. However, UV can cause health hazards (21) and is seldom used for resin sealant now (22). Visible light is commonly used for the polymerization of resin sealant (23).

#### 3.1.2. Fluoride-releasing resin sealant or non-fluoride releasing resin sealant

Resin sealant can also be classified by its fluoride-releasing ability. Fluoride particles such as sodium fluoride or fluoride-releasing glass filler, are incorporated into the material in fluoride-releasing resin sealant (24). It provides an additional way to achieve caries prevention.

#### 3.1.3. Filled resin sealant or unfilled resin sealant

Based on the filler content, we can also classify the resin sealant as filled or unfilled resin sealant. The wear resistance of filled resin sealant is higher than unfilled resin sealant (25). However, the viscosity of sealant also increases with the filler added, therefore leading to a lower ability to penetrate fissures and pits (26).

#### 3.1.4. Hydrophobic resin sealant or hydrophilic resin sealant

Based on the sensitivity to moisture, resin sealant can be classified as hydrophobic or hydrophilic resin sealant. Conventional resin sealant is hydrophobic. Recently, a new generation of hydrophilic resin sealant has been developed by adding multifunctional acrylate monomers with a formulation considering the hydrophilic-hydrophobic balance (27). Hydrophilic resin sealant is believed to be able to overcome the moisture challenge during the operative process of sealant application.

### 3.2. Glass ionomer sealant

Two types of glass ionomer sealants are available, conventional glass ionomer sealant and resin-modified glass ionomer sealant. Conventional glass ionomer sealant is comprised of powders of fluoroaluminosilicate glass and liquids containing polyacrylic acid, tartaric acid and water (28). It sets with the acid-base reactions of powder and liquid when mixed (29). Resin-modified glass ionomer sealant is made by introducing resin-based monomers such as 2-hydroxyethylmethacrylate (HEMA) or UDMA into the conventional glass ionomer (30). The physical properties of resin-modified glass ionomer sealant are improved compared to conventional glass ionomer sealant (31). The resin-modified glass ionomer sealant set with the polymerisation of resin-based monomers followed by acid-base reactions of powder and liquid (32).

### 3.3. Hybrid sealant

Hybrid Sealants include compomer sealant and giomer sealant (33). Compomer is also known as polyacid-modified composite resin. It contains non-reactive inorganic filler particles, reactive silicate glass particles, a polyacid-modified monomer and a photo-initiator (31). Giomer is comprised of pre-reacted glass ionomer (PRG) filler and resin-based monomer matrix (34). Hybrid Sealants are relatively new and not widely used.

Dental sealants can also be classified with colors. Clear, white, and pink are three colors that can be commonly seen for dental sealant. Clear dental sealant allows dentists to see through so that the lesion underneath could be better monitored (35). However, clear sealant itself is more difficult to see, hence potentially leading to a bias repair decision (35).

## 4. Mechanisms of dental sealant in preventing and arresting caries

Placing dental sealant is an effective approach to preventing and arresting caries. Possible mechanisms include the physical barrier created by the sealant, ease of cleaning for the patient, and release of ions that favours remineralisation of the tooth.

### 4.1. Physical barrier

Dental sealant covers the fissures and provides a physical barrier. The cariogenic bacteria are difficult to enter and colonize in the fissure area with the physical barrier. This barrier also prevents food debris from getting into fissures which blocks the nutrition intake and inhibits the growth of dental biofilm (22).

### 4.2. Ease of cleaning

Dental sealant can improve the oral hygiene of patients. Fissures can be deep and narrow. It is difficult or impossible for toothbrush bristles to get into the fissures and clean the area (36). Applying dental sealant can seal the fissures. This turns a plaque retentive occlusal surface into a smoother surface which makes the surface easier to clean (37).

### 4.3. Ion release

Different types of dental sealants can release ions which can prevent and arrest caries. Glass ionomer sealants, hybrid sealants and some resin sealants are fluoride-releasing. Fluoride ion reduces demineralization, promotes remineralization (38) and inhibits the growth of cariogenic bacteria (39). Fluoride-releasing sealant can prevent and stop the progression of caries of the sealed tooth (40). It can also offer protection to the adjacent tooth (41). Some giomer sealants release antimicrobial borate and strontium that inhibit bacterial growth (42). Borate and strontium ions, together with sodium ions released by giomer, could be a buffer to lactic acid (43).

## 5. Effects of dental sealant in preventing and arresting caries in the past 10 years

Studies published in the recent ten years (2012–2022) support dental sealant as an effective means of preventing and arresting caries (Table 1). Because of the heterogeneity of the included studies, we did not perform a meta-analysis in this review. Table 1 shows the caries incidence of different dental sealants. With the results of the studies that have blank control groups, we summarize the preventive fractions are 92% for both resin sealant and glass ionomer sealant at a 6-month follow-up (52); 64% - 88% for resin sealant, and 88% for glass ionomer sealant at an 18-month follow-up (68); 61% for resin sealant and 35% for glass ionomer at a 60-month follow-up (56). Results of hybrid sealant were limited in the literature.

**Table 1:** Summary of randomized clinical trial published from 2012 to 2022.

Author (Year) [Ref]	Duration (month)	Number and age of the Participants (Teeth)	Intervention group (final sample size)	Caries incidence	Sealant retention rate
Antonson et al. (2012) (44)	24	39 children aged 5–9 (78 teeth)	Gp1: Resin sealant (27)	N/A	Gp1:41%
			Gp2: Glass ionomer sealant (27)		Gp2:44%
Bhatia et al. (2012) (45)	12	17 children aged 6–8 (68 teeth)	Gp1: Hydrophilic resin sealant (34)	N/A	Gp1:24%
			Gp2: Hydrophobic resin sealant (34)		Gp2:18%*
Ulusu et al. (2012) (46)	24	173 children aged 7–15 (346 teeth)	Gp1: Glass ionomer sealant (139)	Gp1:3%	Gp1:14%
				Gp2:5%	Gp2:21%*
			Gp2: Resin Sealant (137)		
Bhat et al. (2013) (47)	12	80 children aged 6–9 (320 teeth)	Gp1: Resin sealant with bond (76)	G1:3%	Gp1:82%
			Gp2: Resin sealant (76)	Gp2:3%	Gp2:72%
			Gp3: Hydrophilic resin sealant (76)	Gp3:3%	Gp3:80%
			Gp4: Glass ionomer sealant (76)	Gp4:7%	Gp4:21%
					* **Gp1/2/3 > 4**
Chen and Liu (2013) (48)	24	61 children aged 6–9 (158 teeth)	Gp1: Glass ionomer sealant (75)	Gp1:8%	Gp1:36%
			Gp2: Resin sealant (75)	Gp2:8%	Gp2:72%*
Kumaran (2013) (49)	12	40 children aged 7–10 (160 teeth)	Gp1: Filled resin sealant 1 (38)	N/A	Gp1:66%
			Gp2: Unfilled resin sealant (38)		Gp2:42%
			Gp3: Filled resin sealant 2 (38)		Gp3:29%
			Gp4: Glass ionomer sealant (38)		Gp4:32%
					* **Gp1 > 2/3/4**
Hasanuddin et al. (2014) (50)	12	80 children aged 7–10 (160 teeth)	Gp1: Resin sealant (80)	Gp1:0%	Gp1:68%
			Gp2: Glass ionomer sealant (80)	Gp2:0%	Gp2:24%*
Khatri et al. (2015) (51)	12	34 children aged 6–9 (68 teeth)	Gp1: Hydrophilic resin sealant (32)	Gp1:6%	Gp1:72%
			Gp2: Resin sealant (32)	Gp2:16%	Gp2:50%*
Reddy et al. (2015) (26)	12	56 children aged 6–9 (224 teeth)	Gp1: Filled resin sealant (112)	N/A	Gp1:54%
			Gp2: Unfilled resin sealant (112)		Gp2:64%
Gonçalves et al. (2016) (52)	6	31 children aged 6–8 (114 teeth)	Gp1: Resin-modified glass ionomer sealant (33)	Gp1:3%	Gp1:52%
			Gp2: Resin sealant (35)	Gp2:3%	Gp2:89%
			Gp3: No sealant (28)	Gp3:36%	Gp3: N/A
				* **Gp1/2 > 3**	* **Gp2 > 1**
Haznedaroğlu et al. (2016) (53)	48	40 children aged 7–10 (160 teeth)	Gp1: Glass ionomer sealant (40)	Gp1:10%	Gp1:8%
			Gp2: Resin sealant (56)	Gp2:21%	Gp2:39%*
Al-Jobair et al. (2017) (54)	18	42 children aged 6–9 (168 teeth)	Gp1: Glass ionomer sealant (70)	Gp1:31%	Gp1:26%
			Gp2: Resin sealant (70)	Gp2:27%	Gp2:33%
Askarizadeh et al. (2017) (55)	12	23 children aged 6–9 (92 teeth)	Gp1: Resin sealant (40)	Gp1:3%	Gp1:63%
			Gp2: Hydrophilic resin sealant (40)	Gp2:8%	Gp2:60%
Liu et al. (2018) (56)	60	419 children aged 7–9 (664 teeth)	Gp1: Resin sealant (172)	Gp1:13%	N/A
			Gp2: Glass ionomer sealant (178)	Gp2:23%	
			Gp3: No sealant (165)	Gp3:35%	
				* **Gp1 > 2>3**	
Ntaoutidou et al. (2018) (57)	18	81 children aged 6–12 (218 teeth)	Gp1: Giomer sealant (87)	Gp1:15 %	Gp1:7%
			Gp2: Resin sealant (89)	Gp2: 6%*	Gp2:70%*
Siripokkapat et al. (2018) (58)	12	140 children aged 2.5–5 (280 teeth)	Gp1: Resin sealant (116)	Gp1:8%	Gp1:72%
			Gp2: Giomer sealant (116)	Gp2:20%*	Gp2:15%*
Alsabek et al. (2019) (40)	6	40 children aged 6–9 (80 teeth)	Gp1: Hydrophilic resin sealant (40)	N/A	Gp1:85%
			Gp2: Glass ionomer sealant (40)		Gp2:63%*
Mathew et al. (2019) (59)	12	50 children aged 6–8 (100 teeth)	Gp1: Resin-modified glass ionomer sealant (50)	Gp1:2%	Gp1:78%
			Gp2: Resin sealant (50)	Gp2:0%	Gp2:88%
Prathibha et al. (2019) (60)	12	120 children aged 7–9 (240 teeth)	Gp1: Glass ionomer sealant (111)	Gp1:9%	Gp1:51%
			Gp2: Resin sealant (111)	Gp2:5%	Gp2:76%*
Jaafar et al. (2020) (61)	6	45 children aged 8–12 (90 teeth)	Gp1: Resin sealant (45)	N/A	Gp1:76%
			Gp2: Glass ionomer sealant (45)		Gp2:49%*
Mohapatra et al. (2020) (62)	6	30 children aged 12–15 (120 teeth)	Gp1: Resin sealant (44)	N/A	Gp1:23%
			Gp2: Hydrophilic resin sealant (44)		Gp2:11%
Beresescu et al. (2022) (63)	24	28 children age 6–8 (112 teeth)	Gp1: Hydrophilic resin sealant (56)	Gp1:9%	Gp1:79%
			Gp2: Resin sealant (56)	Gp2:5%	Gp2:80%
Haricharan et al. (2022) (64)	24	180 children aged 6–12 (360 teeth)	Gp1: Resin sealant (180)	Gp1:7%	Gp1:39%
			Gp2: Glass ionomer sealant (180)	Gp2:11%	Gp2:32%
Özgür et al. (2022) (65)	12	57 children aged 6–12 (136 teeth)	Gp1: Resin sealant (50)	Gp1:0%	Gp1:68%
			Gp2: Giomer sealant (50)	Gp2:0%	Gp2:8%*
Reic et al. (2022) (66)	24	80 children aged 6–13 (253 teeth)	Gp1: Glass ionomer sealant (51)	Gp1:27%	Gp1:19%
			Gp2: Filled resin sealant 1 (66)	Gp2:11%	Gp2:46%
			Gp3: Unfilled resin sealant (58)	Gp3:24%	Gp3:21%
			Gp4: Filled resin sealant 2 (58)	Gp4:19%	Gp4:47%
				* **Gp2 > 1/3/4**	* **Gp2/4 > 1/3**
Singh et al. (2022) (67)	12	45 children aged 3–5 (180 teeth)	Gp1: Giomer sealant (90)	Gp1:14%	Gp1:26%
			Gp2: Hydrophilic resin sealant (90)	Gp2:15%	Gp2:76%*
Uzel et al. (2022) (68)	18	50 children aged 7–12 (200 teeth)	Gp1: No sealant (35)	Gp1:26%	Gp1:N/A
			Gp2: Resin sealant 1 (32)	Gp2:9%	Gp2:52%
			Gp3: Resin sealant 2 (33)	Gp3:3%	Gp3:24%
			Gp4: Glass ionomer sealant (33)	Gp4:3%	Gp4:18%
				* **Gp3/4 > 1**	* **Gp1 > 2/3**

* Statistically significant.

## 6. Longevity of dental sealant

The caries preventive effect of dental sealant largely relies on the retention of the sealant. Table 1 shows the retention rates of dental sealants published in the past ten years (2012–2022). For resin sealant, the retention rate ranges from 11% to 89% at a 6-month follow-up (40, 52, 61, 62); from 18 to 88% at a 12-month follow-up (26, 45, 47, 49–51, 55, 58–60, 65, 67); from 24% to 70% at an 18-month follow-up (54, 57, 68); from 21% to 80% at a 24-month follow-up (44, 46, 48, 63, 64, 66). Studies with longer follow-up periods were limited. For glass ionomer sealant, the retention rate ranges from 49% to 63% at a 6-month follow-up (40, 52, 61); from 21% to 78% at a 12-month follow-up (47, 49, 50, 59, 60); and from 14 to 44% at a 24-month follow-up (44, 46, 48, 64, 66). Studies in other follow-up periods were limited. For hybrid sealant, the retention rates range from 8% to 26% at a 12-month follow-up (58, 65, 67). Studies with longer follow-up periods were limited.

## 7. Indications for dental sealant placement based on the evidence

### 7.1. Caries risk of the patient

*Dental sealant is indicated for patients with high caries risk.* The effectiveness of dental sealant was proved to be higher in high caries risk patients when compared with low caries risk patients (37, 69). The benefits of dental sealant exceed the cost when children with high caries risk were targeted (70). The cost-effectiveness of dental sealants is higher in patients with high caries risk can be concluded.

### 7.2. Types of the dentitions

*Dental sealant is suggested for permanent teeth.* A systematic review found that resin sealants placed on occlusal surfaces of permanent molars could reduce caries for up to 4 years when compared to no treatment (22). Another systematic review and meta-analysis also found that resin sealant reduced caries incidence in permanent teeth at a follow-up period of up to 4 years (71). *Dental sealant is suggested for primary teeth*. A randomized clinical trial shows that resin sealant can arrest dentinal caries in primary teeth (72). A systematic review concluded dental sealant had caries preventive effects in primary teeth when compared to no treatment with low-certainty evidence (36). In addition, it is a cost-effective approach to applying dental sealant in primary dentition (69).

### 7.3. Caries status of the tooth

*Dental sealant is effective in preventing caries on sound tooth surfaces.* Studies supported the application of dental sealant in preventing caries on sound tooth surfaces (22, 71). A 3-year randomized clinical trial reported that non-sealed molars exhibited a caries incidence of 98.9% while it was only 25.7% for the sealed molars (73). A systematic review showed that the caries risk of sound teeth sealed with resin sealant occlusally is 76% less within 24 to 48 months follow up, and 85% less in 84 months follow up compared to no treatment (74). *Dental sealant is effective in arresting non-cavitated caries* (40, 61). A systematic review showed that the chance of arresting or reversing non-cavitated occlusal caries with dental sealants is 2–3 times higher when compared with no treatment (75). A clinical study over 44 months suggests that caries without frank cavitation can be arrested with dental sealant (35). Dental sealant was also shown to arrest non-cavitated dentinal occlusal caries 36 months after placement (76). In addition, dental sealants placed on sound surfaces and carious surfaces showed similar survival rates (77). Therefore, dental sealant is effective in arresting non-cavitated caries.

*The effectiveness of dental sealant in arresting cavitated caries is controversial.* A clinical study showed that caries progression was rarely detected for initial caries (ICDAS 2) to moderate caries (ICDAS 4) sealed with resin dental sealant at the 24-month follow-up (78). Dental sealant was found to be effective in arresting ICDAS 3 micro-cavitated caries in permanent molars after a 2-year follow-up (79). However, another study found that dental sealant could arrest non-cavitated caries but not micro-cavitated caries (ICDAS 3) (80).

### 7.4. Types of tooth surfaces

*A dental sealant can be applied on occlusal fissure surfaces.* The effectiveness of dental sealant in preventing dental caries in occlusal surfaces has been approved in a number of studies (22). *Dental sealant could also be applied on axial smooth surfaces.* The application of dental sealant in sound or carious smooth surfaces is also supported by recent studies. A 3.5-year study on children found out dental sealant applied on sound mesial smooth surfaces of first permanent molars can prevent distal caries on primary second molars (81). Dental sealants can also arrest non-cavitated and micro-cavitated caries on proximal surfaces (82, 83).

### 7.5. Eruption status of the tooth

*Dental sealant can be applied on fully erupted or partially erupted molars.* However, the retention rate of the sealant in the partially erupted molars is lower due to saliva contamination and the difficulty in moisture control (44). A randomized 24-month clinical trial revealed that the retention of dental sealants is significantly related to the occlusal eruption stage (84). In this study, researchers found that the retention rate of dental sealants placed on partially erupted teeth was lower than on fully erupted teeth. If a partially erupted molar is to be sealed, glass ionomer sealant is a better choice than resin sealant.

### 7.6. Presence of dental fluorosis

*Dental sealant can be applied on teeth with dental fluorosis.* The choice of sealant material is the key to success. A study found out the total retention of resin sealant (68%) is much higher than glass ionomer sealant (24%) on fluorotic permanent molars of 7- to 10-year-old children after one year (50).

## 8. Operative factors affecting the longevity of dental sealant

The longevity of the dental sealant is affected by several operative factors when applying dental sealant, including moisture control of the operative field, tooth surface preparation, and the application of dental adhesives. These factors should be taken into consideration when applying sealants to enhance the retention and extend the longevity of the sealants.

### 8.1. Moisture control

Moisture control should be achieved when placing dental sealants with no difference for rubber dams, cotton rolls or dental isolation systems. Studies found out the dental sealant placed on saliva-contaminated tooth surfaces would significantly increase the microleakage and reduce shear bond strength (85–88). These provide us with evidence that moisture control is critical when placing dental sealant. There is no difference in the retention rate of dental sealant when using rubber dams, cotton rolls, or dental isolation systems as moisture control. A randomized clinical trial studied the difference in retention of resin dental sealant placed with rubber dam isolation or cotton roll (89). At the 12-month follow-up, no statistical significance was found.Another randomized clinical trial compared cotton roll and the Isolite system (an illuminated dental isolation system with a bite block that provides suction and retraction) as isolation for resin dental sealant and followed for 12 months after placement (90). The result was not statistically significant, indicating the cotton and Isolite system are comparable in placing dental sealant. A randomized clinical trial compared Isolite system, cotton roll and rubber dam at the same time (91). The result agreed with the above two studies. There was no statistically significant difference between these three strategies. However, most patients preferred cotton roll isolation and most patients were less likely to have rubber dam as isolation again (92).

### 8.2. Tooth surface preparation

Tooth surfaces cleaned with pumice and prepared with 37% phosphoric acid for 30 s are suggested.

#### 8.2.1. Surface cleaning

Cleaning the tooth surface is required before applying dental sealant. It is because tooth surfaces are covered by salivary pellicles and products of carbohydrate metabolism which inhibit the penetration of dental sealant into the pits and fissure area (93). Therefore, Sealant placed without tooth surface cleaning was having a low retention rate (94). Resin sealant applied on tooth surfaces brushed with pumice slurry showed a significantly higher retention rate when compared with no treatment and brushing only. Another *in vitro* study compared the microleakage of resin dental sealant placed after dry brushing, pumice slurry cleaning, air polishing and prophylaxis paste polishing (95). Researchers found that air polishing is superior to other treatment. It was followed by pumice; prophylaxis paste and dry brushing. However, air polishing is a risk factor for subcutaneous emphysema when it is not meticulously handled (96). Therefore, using pumice is suggested.

#### 8.2.2. Mechanical preparation with dental burs

Mechanical preparation with burs is not recommended. A clinical study found out the difference in the retention rate of dental sealant between teeth with or without fissurotomy bur preparation was not statistically significant (97). When comparing fissurotomy bur, pumice and no preparation, there was no statistical difference between using fissurotomy bur and pumice (98). However, the resin sealant placed after both treatments were having much lower microleakage than no preparation. An *in vitro* study compared microleakage of resin sealants placed on tooth surfaces treated with round carbide bur, air polishing, air abrasion, pumice, brushing only and longer etching time (99). The microleakage of dental sealant after bur preparation was superior. However, fissures opened with mechanical burs might be more susceptible to caries after the sealant is lost (100).

#### 8.2.3. Mechanical preparation with laser

Current evidence regarding laser as a surface preparation method remains inconclusive. Using laser may not be a better way to increase retention or shear bond strength of dental sealant than acid etching. A randomized clinical trial was done to compare the effect of conventional acid etching and Er,Cr:YSGG laser on the retention rate of dental sealant (101). There was no statistically significant difference between the two groups after 24 months. Another randomized clinical trial comparing Er: YAG, acid etching and Er:YAG before acid etching (102). No statistically significant difference could be found between the groups. Lower shear bond strength was reported when applying dental sealant with Er:YAG instead of acid etching (103). A systematic review and meta-analysis also concluded that using Er:YAG before applying dental sealant is not giving a better retention rate than conventional phosphoric acid etching (104). While there are studies found out Er:YAG laser combined with acid etching can achieve a higher retention rate and less microleakage (105, 106). However, when considering the technical difficulties, the extra cost and the lengthened procedures, the cost-effectiveness of laser is questionable (107).

#### 8.2.4. Mechanical preparation with air abrasion

Air abrasion is also not superior to acid etching in increasing the retention and penetrability of dental sealant. A clinical study compared the retention rate of dental sealants placed with phosphoric acid etching and air abrasion (108). The time allowed for the two treatments was the same. The difference in retention was statistically insignificant. Another study came up with the result that air abrasion prior to acid etching does not increase the retention rate (109). Air abrasion also does not improve the penetrability of dental sealant from the result of an *in vitro* study (110).

A systematic review and meta-analysis comparing acid etching, laser, and air abrasion showed that dental sealant applied with phosphoric acid etching would have a lower microleakage than Er:YAG laser treatment and air abrasion treatment (111).

#### 8.2.5. Chemical preparation with acid etching

Acid etching cannot be replaced by any other tooth surface preparation (100). 37% phosphoric acid etching for resin sealant is suggested (112). A study compared resin dental sealant placed with 15 s, 30 s, 45 s and 60 s acid etching time with 37% phosphoric acid gel (113). Dental sealant applied with 60 s etching time gave out a significantly better shear bond strength. However, when considering the small difference between 30s and 60s, 30s etching time could provide a clinically acceptable result.

#### 8.2.6. Fluoride varnish pre-treatment

Current studies do not support the use of fluoride varnish prior to dental sealant treatment. Use of fluoride does not increase the caries preventive effect and retention of dental sealant. The shear bond strength was adversely affected and the microleakage increased. A clinical study compared dental sealant placed 48 h after fluoride varnish application and dental sealant placed without prior fluoride varnish and no difference in the retention rate was found (114). There was an *in vitro* study compared dental sealant placed 5 min after applying fluoride varnish and sealant placed without fluoride varnish. The result showed that the mean shear bond strength of dental sealant was hugely reduced with the use of fluoride varnish (115). Another *in vitro* study found that applying fluoride varnish 24 h before placing resin or resin modified glass ionomer sealant increases the microleakage (116).

### 8.3. Application of dental adhesive

Current evidence about dental adhesives application prior to dental sealant placement is inconclusive. Several studies showed that dental adhesives increase bond strength and reduce the microleakage of resin sealant (88, 117, 118). Among the dental adhesive systems, the etch-and-rinse dental adhesive system showed a better effect in increasing the bond strength of resin sealant when compared with the self-etching system (118–120). On the other hand, a clinical study found the retention rate of resin sealant with dental adhesives was better than resin sealant without dental adhesives at the 3-month follow-up. However, this difference disappeared at 6-month and 12-month follow-ups (121). Some other studies did not find an enhancement in the retention rate of dental sealant with the addition of dental adhesives (122, 123). Literature shows strong evidence on the operative factors affecting the longevity of dental sealant. Thus, we recommend a step-by-step clinical protocol for sealant placement in Table 2.

**Table 2:** Clinical protocol for placement of dental sealant.

Step	Description [Reference]
1 Isolation	Use cotton rolls, rubber dam or a dental isolation system
	Maintain a clean, dry field for operation (91)
2 Etching	Etch the tooth with 37% phosphoric acid for 30 s (113)
	Avoid acid gets into contact with the tongue.
3 Cleaning	Clean the tooth surface with pumice slurry (94)
	Rinse and dry with 3-in-1 syringe and check for frosty appearance
4 Sealant application	Place dental sealant with a straight probe or thymosin instrument
	Use a straight probe to run through to eliminate any air bubbles.
5 Light curing	Light cure according to the manufacturer's instructions.
	Recommend generally to light cure 20 s.
6 Check occlusion	Check the occlusion with articulating paper.
	Remove high spot / excess and polish.

## 9. Summary

Dental sealants can be classified into resin, glass ionomer and hybrid sealant with corresponding subgroups. Caries risk, type of dentition, caries status of a tooth, type of tooth surfaces, eruption status and dental fluorosis are the considering factors. The effect of dental sealants on preventing and arresting caries is supported by studies published in the recent 10 years. They can effectively prevent and arrest caries by providing a physical barrier, improving the ease of cleaning, and releasing ions. Among different sealants, resin sealant is suggested because of its superior retention and good caries-preventive effect while glass ionomer can be considered when encountering difficult moisture control. Various operative factors, including moisture control, surface preparation and application of dental adhesive, affect the clinical outcomes.
